# Administrative data in pediatric critical care research—Potential, challenges, and future directions

**DOI:** 10.3389/fped.2022.1014094

**Published:** 2022-09-28

**Authors:** Nora Bruns, Anna-Lisa Sorg, Ursula Felderhoff-Müser, Christian Dohna-Schwake, Andreas Stang

**Affiliations:** ^1^Department of Pediatrics I, Neonatology, Pediatric Intensive Care Medicine, and Pediatric Neurology, University Hospital Essen, University of Duisburg-Essen, Essen, Germany; ^2^Center for Translational Neuro- and Behavioral Sciences (C-TNBS), University Hospital Essen, University of Duisburg-Essen, Essen, Germany; ^3^Division of Pediatric Epidemiology, Institute of Social Pediatrics and Adolescent Medicine, Ludwig Maximilian University Munich, Munich, Germany; ^4^University Children's Hospital, Eberhard Karls University, Tübingen, Germany; ^5^Institute for Medical Informatics, Biometry and Epidemiology, University Hospital Essen, University of Duisburg-Essen, Essen, Germany

**Keywords:** pediatric critical care, administrative data, adjustment, confounding, measurement of outcomes, international classification of disease (ICD), diagnose related groups, severity of disease

## Abstract

Heterogenous patient populations with small case numbers constitute a relevant barrier to research in pediatric critical care. Prospective studies bring along logistic barriers and—if interventional—ethical concerns. Therefore, retrospective observational investigations, mainly multicenter studies or analyses of registry data, prevail in the field of pediatric critical care research. Administrative health care data represent a possible alternative to overcome small case numbers and logistic barriers. However, their current use is limited by a lack of knowledge among clinicians about the availability and characteristics of these data sets, along with required expertise in the handling of large data sets. Specifically in the field of critical care research, difficulties to assess the severity of the acute disease and estimate organ dysfunction and outcomes pose additional challenges. In contrast, trauma research has shown that classification of injury severity from administrative data can be achieved and chronic disease scores have been developed for pediatric patients, nurturing confidence that the remaining obstacles can be overcome. Despite the undoubted challenges, interdisciplinary collaboration between clinicians and methodologic experts have resulted in impactful publications from across the world. Efforts to enable the estimation of organ dysfunction and measure outcomes after critical illness are the most urgent tasks to promote the use of administrative data in critical care. Clever analysis and linking of different administrative health care data sets carry the potential to advance observational research in pediatric critical care and ultimately improve clinical care for critically ill children.

## Introduction

Heterogenous patient populations with small case numbers constitute a relevant barrier to research in pediatric critical care. Multicenter prospective studies are associated with financial and logistic challenges and interventional studies in children require very high ethical standards, making retrospective observational studies the prevalent study type in this field (1–4). However, retrospective studies frequently suffer from missing and incomplete data with the potential of information bias.

Disease-specific registries enable high quality research related to conditions of interest, like trauma, resuscitation, (pediatric) critical illness or preterm birth. Several examples from the field of pediatrics show that impactful insights can be drawn from such databases (5–9). Yet, registries bring along high costs for data entry, quality assurance, and maintenance. Unless they are specifically designed for the pediatric population, the documentation may not be able to capture the particularities of pediatric cases.

An alternative that is not yet routinely exploited in pediatric critical care are administrative data, e.g., from national hospital statistics or statutory health insurance companies. The general advantages and disadvantages of using administrative databases in epidemiological, clinical and health research have already been described in previous papers (10, 11). The use of administrative data in the field of pediatric critical care, however, is still rare. This type of data offers otherwise unattainably large numbers of already de-identified cases, ongoing data collection along with standardized coding of diagnoses and procedures, allowing population-based analyses. Administrative data contain detailed information on invasive measures, procedures, and surgeries that are relevant for reimbursement. Highly influential research on severe pediatric trauma has been conducted using administrative data from the National Trauma Data Bank in the US (12, 13). Another example of the successful exploitation of administrative data is the German DRG (diagnosis related groups) data set (14–16) that has recently also been applied for pediatric research (17, 18).

This perspective article will discuss underlying strategies for analysis, advantages and associated pitfalls that apply to all types of administrative data sets, regardless of local regulations and legislations. Due to the authors' origin, the concrete examples given in this paper will be informed by applications using administrative data sets available in Germany. The following paragraph will give a brief overview about the available data sets in Germany and point out particularities of these data sets.

**Figure 1:**
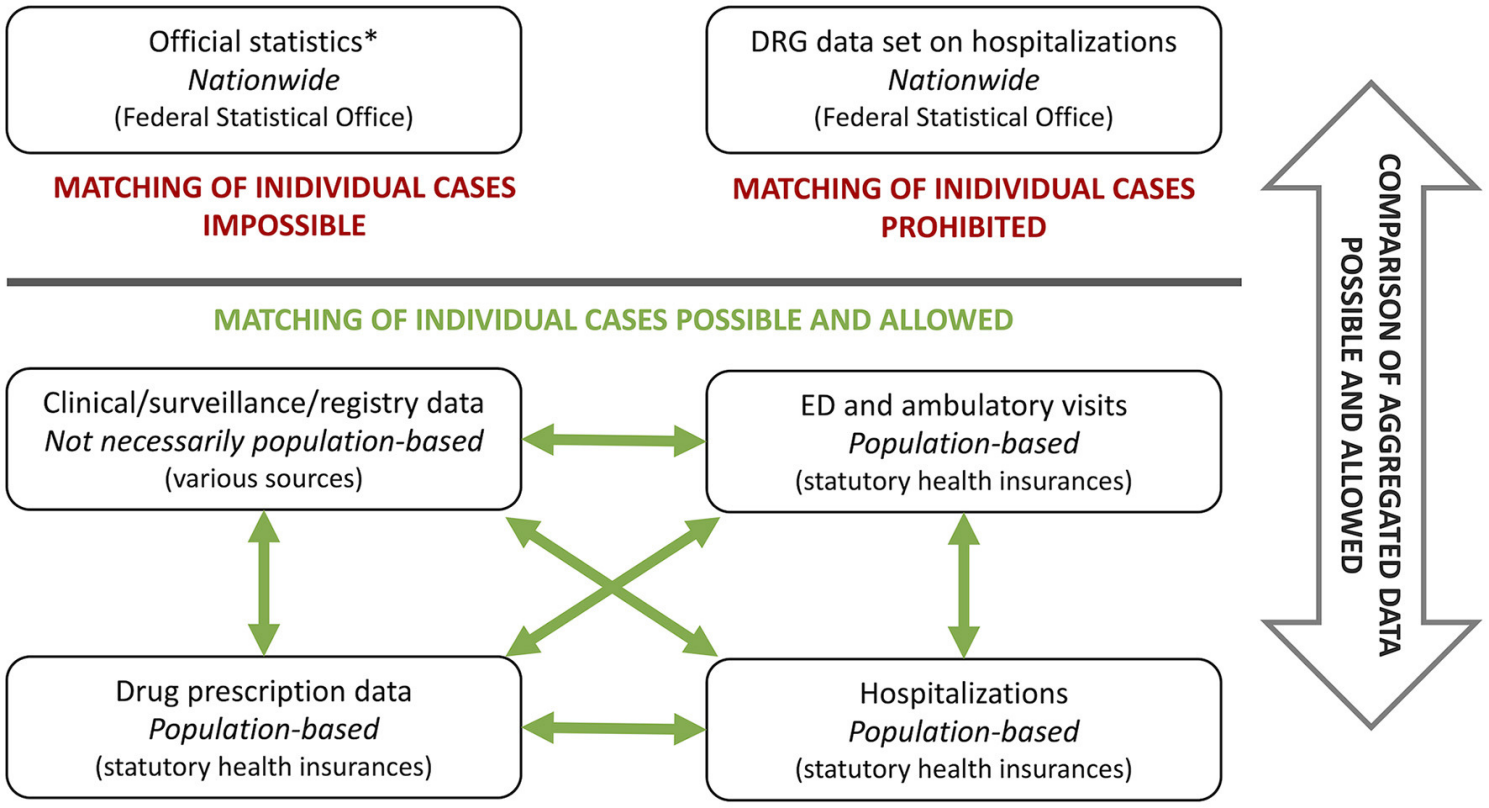
Different types of administrative data sets and possibilities of data linking using Germany as an example. DRG, diagnose related groups; ED, emergency department; *publicly available.

In Germany, the comprehensive use of administrative health care data for scientific research is restricted by data protection laws as well as by insufficiently developed information technology and structural prerequisites. A main shortcoming of the aforementioned is that different types of administrative data cannot be linked with each other on a case basis (Figure 1). Available administrative data sources in Germany are data from hospital information systems, registry data, health insurance data or the DRG data set, which is derived from the hospital remuneration system (Institut für das Entgeltsystem im Krankenhaus, InEK) and provided by the Federal Statistical Office. The DRG data set, which contains all hospitalizations from public hospitals across the country, is completely anonymized and identification of individual cases is strictly prohibited. Data from health insurance companies are anonymized as well, but allow to link several cases of an individual within the same health insurance company via a unique identifier. The over 100 different German health insurance companies store their data in a decentralized manner, making it impossible to link an individual's cases between different companies. Therefore, if an individual changes the health insurance company, the health care information about this person is lost for the researchers.

### Challenges to the use of administrative data by clinicians

Despite the undoubted advantages of administrative data, there are numerous challenges that limit its current use. Knowledge about the availability and characteristics of these data sets is not routinely taught during medical studies or clinical training. From our personal experience, administrative hurdles can be demanding and local data protection regulations may limit in-depth investigations. In order to conduct analyses of administrative data, expertise in the handling of large data sets and epidemiologic methods is indispensable, along with an understanding of the coding system and the clinical content under investigation (10). While epidemiologists have the methodological expertise, highly relevant research questions and interpretation of the analyzed content are most often provided by clinicians who take care for the patients. Thus, close interdisciplinary collaboration to combine methodological and clinical expertise is essential for the success and public health impact of research derived from administrative data sets.

### Limitations of administrative data

An important limitation of administrative data, especially in Germany, is the fact that they are frequently collected for reimbursement purposes rather than being collected for clinical research. Often, clinical details such as severity of diseases, physical and laboratory findings, are unavailable. Comorbidities or complications that are not reimbursement-generating are frequently not coded. However, abundant information is available on procedures and surgeries, which resemble important factors for reimbursement. For this reason, invasive measures can be used to substitute clinical information, e.g., kidney failure indicated by hemodialysis or elevated intracranial pressure suggested by a decompressive craniectomy. Depending on the exact type of data set and local data protection regulations, follow-up information to extract information on outcomes and the possibility to link data from different sources may or not be available.

Further limitations include potential regional differences in coding practices, and selection bias introduced by differences in demographics and morbidities between data sets, e.g., from different health insurance companies. An example that illustrates the strengths and limitations of administrative data is a study on the incidence and outcomes of pediatric inflammatory multisystem syndrome (PIMS) in Germany that used statutory health insurance data (19). In the treatment of this disease, the application of intravenous immune globulins (IVIGs) and steroids constitute the main pillars of therapy with equal importance from a clinical point of view. While the application of IVIGs is relevant for reimbursement and can be extracted from the data set, the application of steroids is not separately reimbursed and therefore cannot be coded.

### Strategies to overcome the limitations of administrative in the assessment of disease severity

An important aspect in the assessment of pediatric critical care cases is the stratification of disease severity to control for confounding. Several classifications and scores have been developed for different patient subpopulations, e.g., trauma patients, neonates, and children (20–23).

#### Scores for pediatric trauma patients

Scores for trauma patients, like the abbreviated injury scale (AIS) and the injury severity score (ISS), are calculated from the initial injury pattern (20, 24). While the original classifications require clinical findings, it has been shown that the AIS and ISS can be retrieved from ICD (international classification of diseases) codes with sufficient reliability also in children (25–31). As a further advancement, mortality risk stratification in trauma patients can be performed directly within administrative data sets by calculating survival risk ratios (SRR) (32). An SRR reflects the survival probability with a certain injury based on the proportional survival of all cases diagnosed with this injury in the data set. The risk of mortality can be estimated from the single worst injury or by multiplication of several SRRs (multiplicative injury score) (33, 34). Both methods, the ICD-based retrieval of AIS and ISS and the calculation of SRRs, have been successfully applied in pediatric trauma patients (12, 13, 30, 34).

#### Scores for pediatric critical care patients

In critically ill non-trauma patients, the degree of organ dysfunction at pediatric intensive care unit (PICU) admission is frequently used to estimate the severity of disease. Common scores are the pediatric index of mortality 2 and 3 (PIM2 and PIM3), pediatric risk of mortality III (PRISM III) score, pediatric logistic organ dysfunction 2 (PELOD-2) score, pediatric multiple organ dysfunction score (P-MODS), and the pediatric sepsis-related organ failure assessment (p-SOFA) score. Importantly, critically ill pediatric patients typically experience a gradual deterioration that finally decompensates, rather than suffering one initial event that inflicts the damage, like trauma patients. Depending on the timepoint of PICU admission, the patient may already have decompensated or still be in the process of deterioration. This implies that the timepoint of PICU admission does not necessarily represent the nadir of a patient's condition.

A vast variety of scoring systems using physiological and laboratory variables has been introduced to assess the mortality risk in critically ill children but their performance in the general PICU population highly depends on characteristics of the studied population (23). Moreover, scoring systems developed in high resource settings do not necessarily perform well in similar settings, but may nonetheless perform well in medium or low resource settings (35–40). Further caution is necessary when subgroups of patients are assessed. None of the currently available scoring systems for organ dysfunction and multiple organ failure encompasses all pediatric critical care settings and—unlike scores in adult intensive care—none has made its way into broad clinical routine (23).

An ICD-based estimation of the degree of acute organ failure entails even more challenges than the clinical estimation at PICU admission described above. Organ replacement therapies can serve as substitute markers for organ failure but it is difficult to determine whether the organ failure is a risk factor or an outcome. For example, renal replacement therapy reflects kidney failure—whether the kidney failure was part of the initial critical condition leading to intensive care admission or a complication acquired during the course of disease can only be deduced from detailed information on the clinical course. Therefore, acute disease severity and the course of acute critical illness will remain difficult to assess from administrative data, even though efforts to develop a classification should be made, nonetheless.

#### Scores for baseline morbidity

Two scoring systems are available to assess chronic conditions. The pediatric complex chronic conditions classification (PCCC) is a well-established tool to extract baseline morbidity from administrative data (41). It takes into account chronic conditions that can be expected to last at least 12 months or until death intervenes. All relevant organ systems are represented, as well as conditions after organ transplants, technology dependence and specific neonatal diseases (41). A newly developed score to assign numerical weights for pre-existing conditions in administrative data is the Pediatric Comorbidity Index (42). It was developed based on the 1-year-hospitalization risk of children displaying any of the index diagnoses, thereby providing a summary score of baseline disease burden.

Though not developed for the critical care setting, applying one of these two classifications to administrative data likely reduces bias, because critically ill children frequently suffer from chronic diseases. Combining these available scores with a future classification of pediatric acute organ dysfunction might narrow the current gap to precisely estimate a patient's medical condition.

### Measurement of outcomes

A frequently measured outcome in critically ill patients is mortality, either in-hospital or within a specific time frame. An important outcome for patients and their families, however, is the functional outcome. Measuring functional outcome in children is even more complex than in adults due to the developmental dynamics during childhood.

So far, administrative data sets provide only surrogate markers of functioning, such as discharge to a rehabilitation facility or hospice. If longer-term follow-up information is available, visits to specialists, attributed levels of care, prescription of drugs and technical devices, and diagnoses coded over time can give cues on sequelae. Databases that contain long follow-up periods of diseased and non-diseased individuals, like health insurance data, enable the simulation of a cohort study, e.g., by following up a birth cohort (43). Hereby the risk of an outcome after a specific disease can be compared to the baseline risk of suffering the outcome of interest in healthy individuals. Another recent example illustrating such an approach is a study that examined the impact of mechanical ventilation during severe respiratory illness on the prevalence of mental disorder diagnoses and psychotropic medication use following hospital discharge in children (44).

Depending on the data set, this may be completely impossible, like in the German DRG data set. This data set is right-censored, meaning that follow-up information stops at the date of discharge and, for example, a 30-day case fatality of a disease cannot be validly estimated. Health insurance data, on the other hand, allow to link an individuals' hospital cases with ambulatory and follow-up cases, thereby providing the possibility to extract the named surrogate outcomes.

### Future directions

Generating scientific results with a high level of evidence in the field of pediatric critical care, which is characterized by rare and complex medical conditions, through prospective interventional studies will continue to be challenging. Population-based administrative health care data will remain a major source to evaluate the epidemiology of diseases and diagnostic or therapeutic strategies that lack evidence.

Growing computational power promotes the use of large amounts of data in biomedical sciences. In this context, administrative data may be an important future component of observational real-world data to evaluate therapies—possibly enhanced by linking these data to clinical, registry or further administrative data in order to compensate specific weaknesses of each data type (Figure 1). Several examples illustrate the potential of linking different types of data sets, including public and administrative data sets: data collected for a German surveillance study on pediatric rare diseases were linked to ICD10-coded discharge diagnosis from hospital discharge data in order to estimate population-based incidence rates of perinatal arterial ischemic stroke using capture-recapture analyses (45). Two other studies linked results from the national hospital data set with drug prescription data obtained from health insurances to quantify the effect of direct oral anticoagulant prescription on gastrointestinal and genitourinary bleedings in Germany (46, 47). To estimate hospitalizations, intensive care unit admissions and deaths due to COVID-19 and PIMS, data from three different sources were combined—a national seroprevalence study, the German statutory notification system and a nationwide registry on children and adolescents hospitalized with either SARS-CoV-2 or PIMS (48). Besides data from public and non-profit sources, industry-acquired data can also be combined with the aforementioned, as recently carried out to investigate the effect of social distancing on antibiotic use in children hospitalized for status asthmaticus (49).

These collaborative studies performed by clinicians and epidemiologists show that the use of administrative health care data and the application of advanced epidemiological methods generates impactful results that are not achievable by clinical trials alone. Basic methodological training of clinician scientists to increase the awareness for available administrative health care data and epidemiologic methods should constitute the first step toward closer collaboration. Joint efforts by pediatric intensivists and epidemiologists have the potential to promote the exploitation of real-world data to enhance clinical research and ultimately improve clinical care for critically ill children.

However, increasing use of administrative health care data should also be accompanied by validation studies of these data. In Germany, for example, relevant differences exist regarding demographics and morbidities between health insurance companies, possibly limiting the generalizability of acquired results (50). Unfortunately, data protection regulations in Germany make a representative validation study of German hospital data or data from health insurance companies virtually impossible.

## Conclusion

Research in pediatric critical care faces many obstacles. Detailed clinical information can only be obtained at high expenses and is often associated with limited case numbers. Clever analysis and linking of different administrative health care data sets carry the potential to advance observational research in pediatric critical care. Nonetheless, efforts to enable the estimation of organ dysfunction and measure outcomes are essential to pave the way for meaningful results.

## Data availability statement

The original contributions presented in the study are included in the article/supplementary material, further inquiries can be directed to the corresponding author.

## Author contributions

NB: conceptualized the work and wrote the initial manuscript. AS, A-LS, CD-S, and UF-M: critically revised the manuscript for important intellectual content. All authors agree to be accountable for the content of the work.

## Conflict of interest

The authors declare that the research was conducted in the absence of any commercial or financial relationships that could be construed as a potential conflict of interest.

## Publisher's note

All claims expressed in this article are solely those of the authors and do not necessarily represent those of their affiliated organizations, or those of the publisher, the editors and the reviewers. Any product that may be evaluated in this article, or claim that may be made by its manufacturer, is not guaranteed or endorsed by the publisher.
